# Achieving the Sustainable Development Goals for water and sanitation in Indonesia – Results from a five-year (2013–2017) large-scale effectiveness evaluation

**DOI:** 10.1016/j.ijheh.2020.113584

**Published:** 2020-09

**Authors:** Mitsunori Odagiri, Aidan A. Cronin, Ann Thomas, Muhammad Afrianto Kurniawan, Muhammad Zainal, Wildan Setiabudi, Michael Emerson Gnilo, Chander Badloe, Tri Dewi Virgiyanti, Imran Agus Nurali, Laisa Wahanudin, Aldy Mardikanto, Paul Pronyk

**Affiliations:** a UNICEF, Indonesia; b UNICEF, Zimbabwe; c UNICEF, New York, USA; d National Development Planning Agency (Bappenas), Government of Indonesia, Indonesia; e Ministry of Health, Government of Indonesia, Indonesia

## Abstract

**Background:**

Access to safe sanitation and the elimination of open defecation are pre-conditions for improved child health and nutrition and wider achievement of the Sustainable Development Goals (SDGs). While Indonesia has a solid policy framework, the country ranks third globally in terms of numbers of people practicing open defecation.

**Objectives:**

Our aim was to assess the effectiveness of a five-year strategy to reduce open defecation through accelerating implementation of the national sanitation program across districts receiving variable levels of external support.

**Methods:**

Among three provinces with poor sanitation program performance, districts were selected to receive one of three levels of external support. High intensity districts (n = 6) benefitted from enabling environment strengthening support including political and social mobilization, direct capacity development, and efforts to strengthen planning, budgeting, monitoring and supervision; learning districts (n = 16) benefitted from cross-district learning opportunities and political mobilization through provincial government advocacy efforts; and comparison districts (n = 58) were monitored under routine program conditions. Outcomes included open defecation free (ODF) status and new toilet facility construction and were assessed through village level monitoring systems across all districts. Negative binomial regression and multivariate analysis were used to assess associations between levels of intervention intensity and outcomes.

**Findings:**

Among districts receiving high-intensity external support improvements in political commitment, planning, coordination, financing, monitoring and supervision were observed. Relative to comparison districts, high intensity districts were more likely to be ODF (aRR 4.65, CI 2.12–10.20) with greater increase in household toilet coverage (aRR 11.15 CI 1.04–119.82). Weaker non-significant associations with ODF were observed among learning districts relative to comparison districts.

**Interpretation:**

Efforts to strengthen provincial and district government capacity to implement sanitation programming in Indonesia can yield substantial improvements in outcomes in a relatively short period of time.

## Highlights

• The effectiveness of five-year sanitation strategies in Indonesia was assessed.
• In high-intensity districts improvements in government systems were observed.
• High-intensity districts were more likely to be open defecation free.
• Sanitation outcomes can be improved when government systems are strengthened.

## 1 Introduction

Poor water, sanitation and hygiene (WASH) are major contributors to diarrheal disease and preventable child deaths (Cairncross et al., 2010) - accounting for at least 600,000 deaths of children under 5 years annually (Cronin et al., 2017a; Wolf et al., 2018). Low WASH coverage is associated with an additional range of adverse health-outcomes including intestinal parasitosis (Lin et al., 2018), stunting (Torlesse et al., 2016) and maternal deaths (Benova et al., 2014). Adverse consequences are felt disproportionately among women - with reduced dignity, privacy and security (Cronin et al., 2015; Sommer et al., 2015) alongside opportunity costs related to income loss from preventable illness (Laughland et al., 1993).

While access to improved water witnessed substantial gains globally since 1990 – from 76% to 91%; access to improved sanitation has lagged behind – increasing from 54% to 68% by 2015. Furthermore, the WASH targets for the Sustainable Development Goals (SDGs) are more ambitious, aiming for safe and affordable water alongside safely managed sanitation including open defecation free (ODF) communities (World Health Organization (WHO) and the United Nations Children's Fund (UNICEF), 2017).

Globally, an estimated 673 million people still defecate in the open (United Nations Children's Fund (UNICEF) and World Health Organization (WHO), 2019). Indonesia ranks third in the world with over 25 million people not using sanitation facilities (United Nations Children's Fund (UNICEF) and World Health Organization (WHO), 2019; National Bureau of Statistics Indonesia (BPS), 2018). Despite recently achieving middle-income status, nearly one third of Indonesian households live below or dangerously close to the poverty line (World Bank Indonesia, 2018). Nearly one in 30 children dies before reaching primary school – with figures as high as one in 15 in the eastern part of the country (National Population and Family Planning Board (BKKBN)Statistics Indonesia (BPS) et al., 2018). While substantial decline in diarrhea mortality rate in Indonesia has been observed since 1990 because of progress in sanitation (Troeger et al., 2019), highlighting its contribution to public health programming, in the post-neonatal period, diarrhoea and pneumonia still remain the most common causes of death (Ministry of Health and Indonesia, 2016). Among Indonesia's 34 provinces and 514 districts, just 58 districts and one province have achieved ODF status (Ministry of Health and Indonesia, 2020). Although just over 9% of households practice open defecation (National Bureau of Statistics Indonesia (BPS), 2018), approximately half of population resides in communities where open defecation is prevalent (United Nations Children's Fund (UNICEF) and World Health Organization (WHO), 2019). Furthermore, while 82% of people have access to improved drinking water, studies suggest as little as 9% drink water from a safely-managed source – a key SDG target for water quality (Cronin et al., 2017b). In Indonesia, elimination of open defecation in communities is crucial in reducing environmental fecal contamination and optimize health and nutrition gains (Cronin et al., 2017a), however, poor sanitation facilities are widely being used, posing serious risks of fecal contamination in the environment such as groundwater for drinking (Cronin et al., 2017b).

To address these gaps, the Government of Indonesia has recently included the target of universal access to basic sanitation and safe water for all within its National Development Plan of 2020–2024. The country has committed to eliminating open defecation and ensuring 90% of households have access to improved sanitation by 2024, of which 15% will be safely-managed. To achieve these ambitious aims, the national sanitation program outlines five key intervention areas for community-based WASH support: use of toilets; handwashing with soap; safe storage and handling of drinking water; and effective solid and liquid waste management.

While the national government is the custodian for this agenda, a range of structural challenges have constrained implementation including the highly-decentralized nature of governance; institutional fragmentation *vis a vis* leadership for WASH-sector activities, with local capacity gaps at the provincial and district levels; underbudgeting and underspending in the sector; and the absence of high-quality costed operational research to inform planning and budgeting for models that work at scale. These barriers are even more critical given the SDG imperative of universality and ensuring the equity, quality and sustainability of services.

This paper profiles learning from a five-year effort to support and scale Indonesia's national WASH program. We conducted a large-scale effectiveness evaluation (Victora et al., 2011) to respond to several key objectives: to document the implementation of programmatic and system strengthening interventions among districts with varying levels of external support across three diverse provinces; to assess progress towards eliminating open defecation; and to generate lessons to inform program support requirements for replication and national scale in response to the Indonesian government's ambitious SDG WASH sector targets.

## 2 Methods

In 2012, the Government of Indonesia, with the support of UNICEF, launched a five-year sanitation and hygiene acceleration plan within three eastern provinces of Nusa Tenggara Timur (NTT), South Sulawesi and Papua (see locations in Supplemental Material). The three focus provinces broadly reflect areas in eastern Indonesia with a range of sanitation coverage (average or poor) and a large burden of chronic childhood undernutrition (stunting prevalence ranging from 40 to 52%) (National Institute of Research and Development and Ministry of Health, 2013). The program offered *high-intensity support* to districts (n = 6) within these three provinces with the aim of eliminating open defecation through accelerating implementation of intervention areas outlined in the national sanitation program in line with the national priority (Box 1). This included ground-level support to improve the capacity of government staff, enhanced political mobilization and social advocacy, support for sector coordination, planning, monitoring and supervision, and regular feedback (United Nations Children's Fund (UNICEF), 2015). Additional *learning districts* (n = 16) in these same provinces received more limited support which included political mobilization through advocacy efforts of provincial WASH working groups, provincial trainings, review meetings and cross-district learning opportunities. A remaining group of *comparison districts* (n = 58) were monitored while receiving no additional support outside routine efforts to implement the national sanitation program. High-intensity districts were purposively selected in close consultation with provincial governments, targeting average to relatively poorly performing districts where strong international development partners were not present, while learning districts were chosen by provincial governments based on their commitment to participate in the programme. Levels of policy and program intensity for the three groups of districts are summarized in Table 1. The program was implemented by the Indonesian Ministry of Health (MoH) with a strong support from the National Development Planning Agency (Bappenas) in collaboration with the various sub-national government stakeholders at the community, district and provincial levels. High-intensity and learning districts included both rural and urban contexts (i.e. rural and urban villages known as *Desa* and *Kelurahan*, respectively).

Box 1 **Sanitasi Total Berbasis Masyarakat (STBM) or Community Based Total Sanitation in Indonesia.**

The Government of Indonesia's STBM (Sanitasi Total Berbasis Masyarakat or Community-based to Total Sanitation program) has focused on the need for stronger interpersonal approach to generating and sustaining demand of households in rural Indonesia around five key WASH issues: stop open defecation, washing hands with soap, safely storing and handling drinking water and then solid and liquid waste management. The approach is global best-practice strategy for enhancing community awareness and ownership on the importance of improved sanitation and hygiene (United Nations Children's Fund (UNICEF), 2016).

STBM is managed at national level by Ministry of Health and implemented at local level through health facility-based by outreach workers known as sanitarians. Coordination with other sanitation initiatives is managed through a WASH working group at Province and District level and chaired by the local Development Planning Agency (BAPPEDA) or Regional Secretariat Office (Sekretariat Daerah). Implementation of STBM is the responsibility of the District Health Office (DHO), while Provincial Health Office (PHO) plays a monitoring and facilitation role to oversee district performance together with relevant capacity building activities. At sub-district level, sanitarians work closely with village leaders, health volunteers, religious leaders and other relevant stakeholders to effectively mobilize communities. The process follows Community-led Total Sanitation (CLTS) principles whereby sanitarians help to raise awareness about open defecation among the community to trigger collective behaviour change with the aim of eliminating open defecation. Sanitarians also provide technical advice such as affordable sanitation technology options and conduct regular follow-up visits with local health volunteers for the purpose of monitoring progress – which in Indonesia includes the use of real-time SMS tools and web-based monitoring platforms.

Alt-text: Box 1

**Table 1:** Interventions by District typology.

District typology	Intensity level and financial support	Description of interventions and support
*High intensity districts (n = 6)*	High	• Support to a WASH working group coordination platform • Training of Government frontline workers • Support to monitoring visits of frontline workers • Direct advocacy to local leaders (village heads, religious leaders, women's development groups) • Direct advocacy to District heads for strengthening policy and financing • Support to micro-planning initiatives • Direct strengthening of the monitoring and oversight mechanisms • Regular review and follow-visits with District Health and Planning Departments.
*Learning districts (n = 16)*	Medium to Low	• Inclusion of indirect Districts at Province level reviews and trainings • Advocacy for strengthened Enabling Environment interventions • Cross-learning events
*Comparison districts (n = 58)*	None	• Advocacy for acceleration of the national sanitation program at the Province level

Baseline conditions obtained through a formative study in the high-intensity districts are previously described (Hirai et al., 2018). Briefly, while most households had access to improved water source access (86%, range 70–95), just 2/3 had access to improved sanitation (62% range 28–85) with a third of households practicing open defecation (32%, range 14–62). Just over half of respondents performed handwashing after defecation (53%, range 21–87). General demographic and other baseline conditions based on national household surveys in high-intensity, learning and comparison districts can be found in the Supplemental Material (Tables S1).

Table 2 lists program performance and outcome indicators with their definitions and data sources. Data sources for the number of ODF villages, poverty level and open defecation prevalence were collected through national household surveys (Susenas, National Bureau of Statistics) alongside a mobile phone-based monitoring platform (Ministry of Health). Assumptions and approaches to data collection for all indicators can be found in the Supplementary Material.

**Table 2:** Primary outcome and key performance indicators, their definitions and data sources.

Indicators			Definition	Data source	Focus districts
**Primary outcome indicators**					
1	Open defecation free (ODF) villages		Proportion of ODF villages that were certified according to MoH guidelines and reported in the SMS based database in the project period.	Mobile platform data (collected by MoH)	High-intensity, learning and comparison districts
2	Household toilets		Proportion of households building a toilet during the project period. For more details and definitions, see the Supplemental Materials.	Mobile platform data (collected by MoH)	
***Key performance indicators***					
1	Sustained toilet use behavior	Village-level average of proportion of households reporting consistent toilet use in sampled ODF villages in high intensity districts 1–3 years after verification. Toilet usage was measured by a combination of observation and self-reporting.	Programme endline survey (collected by AAN/UNICEF, 2017)	High-intensity districts	
2	Sanitation access among the poorest	Proportion of households gaining access to toilet among the poorest below 40% based on the national threshold	(1) National socio-economic survey SUSENAS (National Bureau of Statistics, Indonesia) (2) Programme endline survey (AAN/UNICEF, 2017)	High-intensity districts	
3	Effectiveness of implementation of STBM, the national community-led sanitation programme	Proportion of villages achieving ODF after community mobilization was initiated through the national community-led sanitation programme in the project period.	Mobile platform (collected by MoH)	High-intensity and learning districts	
4	Leveraged government resources for scaling up in project districts	Number of project districts with plans and budgets for scaling up the national community-led sanitation programme, STBM	(1) Local government documents (2) Programme monitoring documents (collected by UNICEF)	High-intensity and learning districts	
5	Diffusion of the project impact into non-project districts	Number of non-project districts with plans to implement the national community-led sanitation programme		Comparison districts	

The performance of each district was compared based on levels of support received across the three district groupings relative to the primary outcomes of interest – percentage point increase in ODF villages and newly constructed household toilets per district over the programme period (2013–2017). Negative binomial regression was used to assess associations between levels of intervention intensity and key sanitation outcomes. Clustering at the provincial and district levels were accounted for with the application of robust standard error estimations. Adjustments were made for a range of confounders including geographic location, proportion of people living below district-specific poverty line, and baseline proportion of (1) non-ODF villages within each district for the ODF village outcome together with the number of OD villages per district as an offset variable, and (2) OD households within each district for the household toilet outcome together with the number of households per district as an offset variable, to model observed rate/proportion of outcomes. In the case of Papua, due to the absence of provincial data on number of villages in one district (Yalimo), adjustments were inferred from population estimates and the best available data on the average number of dwellers per village. All analyses were conducted in SPSS ver. 22 (SPSS Inc., Chicago, IL).

A complementary costing assessment was conducted using a top-down budgeting approach to estimate the overall programme costs, contribution from households and unit costs of toilets. This approach is commonly used in WASH program costing due to the inability to disaggregate costs by categories of interest (See Supplemental Material). (Crocker et al., 2017).

## 3 Results

Table 3, Table 4 profiles shifts in intervention coverage and performance indicators over the project duration.

**Table 3:** Primary outcomes and adjusted rate ratio (aRR).

Typologies	Primary outcome indicators	Percentage point increase at the end of the programme	aRR (95CI)
Comparison districts	ODF villages	13.8%	Ref.
	Household toilets	8.5%	Ref.

Learning districts	ODF villages	18.6%	1.75 (0.94–3.27)
	Household toilets	5.5%	0.97 (0.30–3.10)

High-intensity districts	ODF villages	42.7%	4.65 (2.12–10.20)
	Household toilets	19.2%	11.15 (1.04–119.82)

**Table 4:**
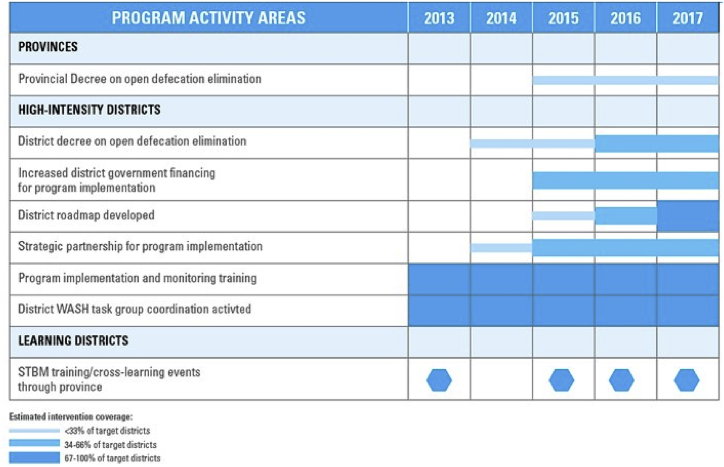
Changes in intervention coverage over the programme duration.

### 3.1 District sanitation performance over the intensity levels of support

**Fig. 1:**
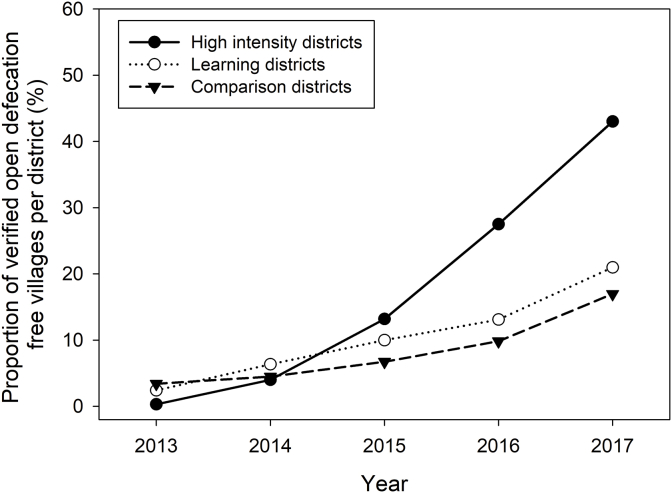
Proportion of cumulative open defecation free (ODF) villages per district over three levels of programming intensity groups (high intensity, learning and comparison districts) in all three programme provinces.

Proportion of ODF villages per district in three district groupings were plotted to examine temporal pattern in the progress of the ODF outcomes during the programme period (Fig. 1).

In the multi-variate analysis, associations were observed between levels of programme intensity and ODF village status. High intensity districts outperformed comparison districts (aRR 4.65 CI 2.12–10.20); with weaker associations observed in learning relative to comparison districts (aRR 1.75, 95%CI 0.94–3.27).

New toilet construction varied similarly by intervention intensity. Over the program period, 19.2 percentage point increase in household sanitation coverage were observed in high intensity districts (aRR 11.15, 95%CI 1.04–119.82), 5.5 percentage point increase in learning districts (aRR 0.97, 95%CI 0.30–3.10) and 8.5 percentage point increase in comparison districts.

### 3.2 Programme performance as measured by key performance indicators

For the sustained toilet use behaviour measurement, high levels of sustainability were observed in sampled ODF villages of high intensity districts even 1–3 years after ODF verification (village-level average: over 94%). Levels of sustainability varied between villages, ranging from 75.6 to 100%.

Among the poorest 40% of households the increase in households owning a private toilet ranged from 8 to 27% in high intensity districts.

Over the project period, the observed proportion of villages achieving ODF in high-intensity districts and learnings districts after community mobilization was initiated was 41.7% and 36.7%, respectively (χ, *P* = 0.011).

Effective planning and budgeting were assessed through provincial and district administrative documentation which suggested 100% of high intensity districts and 75% of learning districts prioritized budgetary allocations for enhanced sanitation activities. Furthermore, 48% of comparison districts were found to have plans to implement or scale the directives of the national sanitation program as a result of varying levels of advocacy efforts derived from the programme. Types of support and contributions to non-project districts varied by province and included monitoring and evaluation strengthening support through provincial WASH task groups, creation of cross-learning platforms, support to a strategy development for acceleration of STBM implementation at provincial level (See Supplemental Material).

### 3.3 Overall programme costs and leveraged household contribution

**Fig. 2:**
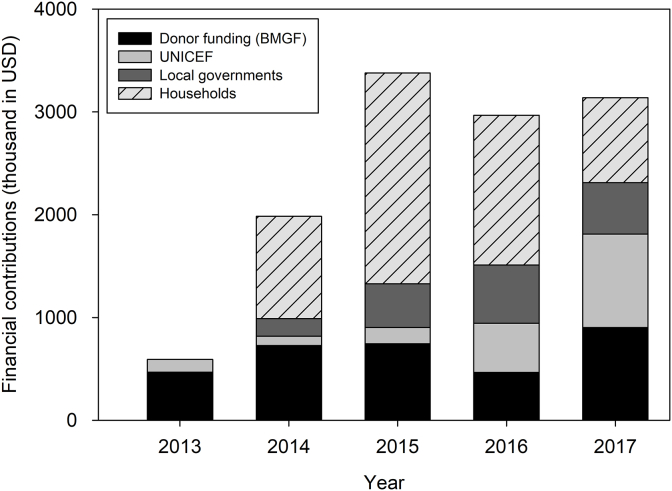
Overall programme expenditure of the five-year support (donor fund and UNICEF) and financial contributions from local government (high-intensity districts) and households (high-intensity and learning districts). Household contribution was estimated based on the number of toilets built by households and unit cost. See Supplemental Material for details.

In the 5-year programme, increase in prioritized budgetary allocations for enhanced sanitation activities together with household contribution as measured by toilet construction was observed (Fig. 2). Households contributed to almost equal amount of the overall programme cost by constructing toilets. The overall unit cost of toilets leveraged by this program was $21.50 per toilet with a considerable variation between programme locations, raining from $6 to $103 per toilet. The wide range is the result of different levels of local government capacities and remoteness and availability of local building materials as well as varied financial capabilities of households in some areas.

## 4 Discussion

Achieving access to adequate and equitable sanitation and hygiene and ending open defecation are critical SDG-related priorities in Indonesia (Bappenas (Indonesia Ministry of National Development Planning) and the United Nations Children's Fund, 2017). This assessment profiles a five-year program that examines several support modalities for accelerating progress towards these targets. Given the country's highly decentralized system, efforts focused on sustainable strategies for improving district government capacity to manage and implement directives outlined in the national sanitation program. Over the program period, gains across a number of program performance areas were observed among districts receiving enhanced support – including demonstrated political commitment, the development of a roadmap and coordination mechanism, increased government financing, strategic partnerships and improved supervision, monitoring and feedback mechanisms. Major improvements were also observed in the achievement of key sanitation outcomes in these districts – open defecation free status and the construction of sanitation facilities. More limited gains were observed among districts benefitting from cross-district learning opportunities and higher-level advocacy efforts.

Given Indonesia's geographic diversity and high-levels of poverty, progress towards sanitation targets has been elusive. Our experience suggests that efforts to strengthen and support existing government systems and particularly those at subnational level in conjugation with strong political commitment can yield substantial benefits in a relatively short period of time even in challenging areas. Additionally, with government system strengthening efforts, government financial contribution could leverage household investments effectively. In the context of the SDG target and timeline, using the average results achieved here over a five-year period and extrapolating to all districts in terms of OD, only a timeline of 4 years would be required to achieve ODF in all villages of the country. However, due to limited national government resources being unable to provide high intensity support to all districts in Indonesia, improving peer-to-peer learning systems might be equally important to support learning districts performance. Finally, the high-level of sustainability observed suggests temporary intensification of resource and support injections may be sufficient to achieve durable gains (Odagiri et al., 2017). In contrast to the study, a recent evaluation of a randomised control trial in Indonesia highlighted the challenges for scaling up by local government (Cameron et al., 2019), reaffirming the importance of government system strengthening. Overcoming challenges of inadequate resource allocation and ensuring effective prioritization and inter-ministerial coordination within the WASH sector will be critical.

Our work echoes the experience elsewhere in the world, notably India's Swachh Barat program, and Bangladesh's national community-led total sanitation (CLTS) program, and Thailand's safe sanitation initiative. In all instances, substantial progress in eliminating open defecation was achieved through the creation of a national sanitation movement involving unified political commitment across ministries and coherent policies to engage communities through a range of stakeholders, including with private financing to enhanced access to safe sanitation (Government of India, 2017; Graham and Selendy, 2011; Hanchett et al., 2011).

Though all three countries achieved notable progress, different approaches were used in each. In India's Swachh Barat program, the government invested over 20 billion USD in sanitation over past five years resulting in a nearly 50% reduction in open defecation (Government of India, 2017). In this model, subsidies were offered to households primarily for construction; however, as in Indonesia's experience and elsewhere, investment in the process accelerated both progress and sustainability. In Bangladesh, sustained national government championing of sanitation was a cornerstone for effectively achieving national ODF status and sustaining this outcome. While local government leadership was a challenge in the Bangladesh context, sustained financing at local levels was one strategy for bolstering subnational governments and providing effective mechanisms for reaching the poorest (Hanchett et al., 2011). The fact that the CLTS approach was championed in the country and that significant national and international engagement pushed sanitation progress forward were extremely critical elements. This is in contrast to Thailand's progress on sanitation which was done entirely by Government – with high levels of political commitment at all levels to develop and disseminate locally appropriate approaches (Graham and Selendy, 2011).

The study had several limitations. First, use of government programme routine data may have potential data quality challenges and bias. Particularly, household toilet construction data from MoH may not capture potential slippage (i.e. reversion to open defecation or non-consistent use of a toilet). However, our ODF sustainability surveys demonstrated high levels of consistent toilet use when household members are at home once ODF status has been achieved (Odagiri et al., 2017). Moreover, to address this issue, we made efforts to gather the best available data from multiple sources coupled with our programme monitoring data which assesses coverage and change over time in high-intensity and learning districts, using the district as the unit of analysis. Secondly, there are limitations of the observational design applied in the study. For example, it is plausible that the presence of other development partners and their support confounded the outcomes of interests in the comparison districts. However, with a high number of comparison districts (N = 58) this effect might be diluted. Importantly, intervention-comparison evaluation designs of real-world programmes going to scale were not feasible in this context, and required more pragmatic assessment methods as described elsewhere (Victora et al., 2011). Finally, in the financial analysis, budget allocations from learning district government were not quantified. Hence, the overall government financial contribution was likely to be underestimated.

In summary, we suggest that Indonesia's SDG targets for sanitation, while ambitious, are within reach. Lessons learned from this effort suggest high-level advocacy and enhanced political will, coupled with strategic technical and resource inputs at the subnational level, including strengthening peer-to-peer learning between local governments in sanitation sector, are key ingredients for accelerating progress.

### 4.1 Research in context

#### 4.1.1 Evidence before study

Despite the importance of sanitation interventions to disrupt the fecal-oral transmission of pathogens in low-and-middle income countries, there is limited evidence on how to deliver sanitation programming at scale. A mixed-method systematic review of Community-Led Total Sanitation (CLTS) published in 2018 by Venkataramanan and others reported that the evidence base documenting drivers of programme effectiveness was weak; which knowledge gaps in relation to WASH-related interventions that are effective across wide areas that describe both outcomes alongside a more detailed description of intervention processes. Similarly, while a systematic review and meta-analysis of effects of sanitation interventions on child health and nutrition, there was relatively poor documentation of critical sanitation outcomes (coverage, use, sustainability) and limited research on factors contributing to successful program implementation.

#### 4.1.2 Added value of this study

We assessed the effectiveness of large scale five-year sanitation strategies in eastern part of Indonesia, on strategies to enhance implementation of the existing national programme. We documented substantial progress across key systems-related barriers related to political leadership, coordination, planning, financing, implementation and monitoring, with strong effects on increased percentage of ODF villages and newly constructed toilet facilities relative to districts receiving business-as-usual external support.

#### 4.1.3 Implications of all the available evidence

Globally, 673 million people still practice open defecation, and acceleration of the progress is urgently needed to end open defecation by 2030. Our results together with global evidence suggest that substantial improvements in sanitation outcomes can be achieved in a relatively short period of time when existing local government systems and political commitment are adequately strengthened. A mixture of high-intensity and learning district approach has the potential to systematically improve local government capacity and strengthen political commitment with limited national government resources at scale.

## Contributors

MO, AAC, MEG, CB, AT and PP conceptualized the study. MO, AAC, MAK, MZ, and WS contributed to data collection. MO performed statistical analysis. MO, AAC, AT and PP led the drafting of the manuscript. All authors revised the manuscript, provided critical input to the interpretation of the data, and approved the final version.

## Disclaimer

The findings and conclusions expressed are those of the authors and do not necessarily reflect the views of UNICEF or the United Nations or the other institutions to which the authors are affiliated..
