# Supplementary material for: Achieving the Sustainable Development Goals for water and sanitation in Indonesia – Results from a five-year (2013–2017) large-scale effectiveness evaluation
Source: Int J Hyg Environ Health. 2020 Sep;230:113584. doi: 10.1016/j.ijheh.2020.113584 (PMC7607394; doi:10.1016/j.ijheh.2020.113584)
Supplement: Multimedia component 2 [file mmc2.docx]

**Supplemental Material**

**Achieving the Sustainable Development Goals for water and sanitation in Indonesia – results from a five-year (2013-2017) large-scale effectiveness evaluation**

1. Study sites baseline conditions and locations

**Table S1.** General demographic and baseline conditions of high intensity, learning and comparison districts (data source: provincial government data)

| Province | Levels of external support | Average N of villages per district | Average N of households per district | Average poverty rate (%) | Proportion of households practicing open defecation at baseline (%) | Proportion of open defecation free villages at baseline (%) |
| --- | --- | --- | --- | --- | --- | --- |
| South Sulawesi | High intensity districts | 109 | 60867 | 11.2 | 21.1 | 0.6 |
|  | Learning districts | 140 | 75796 | 10.9 | 15.8 | 3.7 |
|  | Comparison districts | 124 | 89107 | 10.1 | 18.8 | 0.6 |
|  |  |  |  |  |  |  |
| NTT | High intensity districts | 166 | 47148 | 27.3 | 29.3 | 0.0 |
|  | Learning districts | 145 | 54112 | 20.5 | 24.9 | 2.8 |
|  | Comparison districts | 149 | 50246 | 23.9 | 20.9 | 11.4 |
|  |  |  |  |  |  |  |
| Papua | High intensity districts | 144 | 29149 | 14.7 | 17.6 | 0.0 |
|  | Learning districts | 132 | 38482 | 22.9 | 12.6 | 0.8 |
|  | Comparison districts | 130 | 25007 | 30.7 | 47.2 | 0.0 |
|  |  |  |  |  |  |  |
| Overall | High intensity districts | 134 | 51008 | 17.2 | 23.3 | 0.3 |
|  | Learning districts | 139 | 56382 | 17.8 | 16.9 | 2.4 |
|  | Comparison districts | 134 | 50778 | 22.8 | 31.4 | 3.5 |

**Figure S1.** Location of three programme provinces and districts in Indonesia


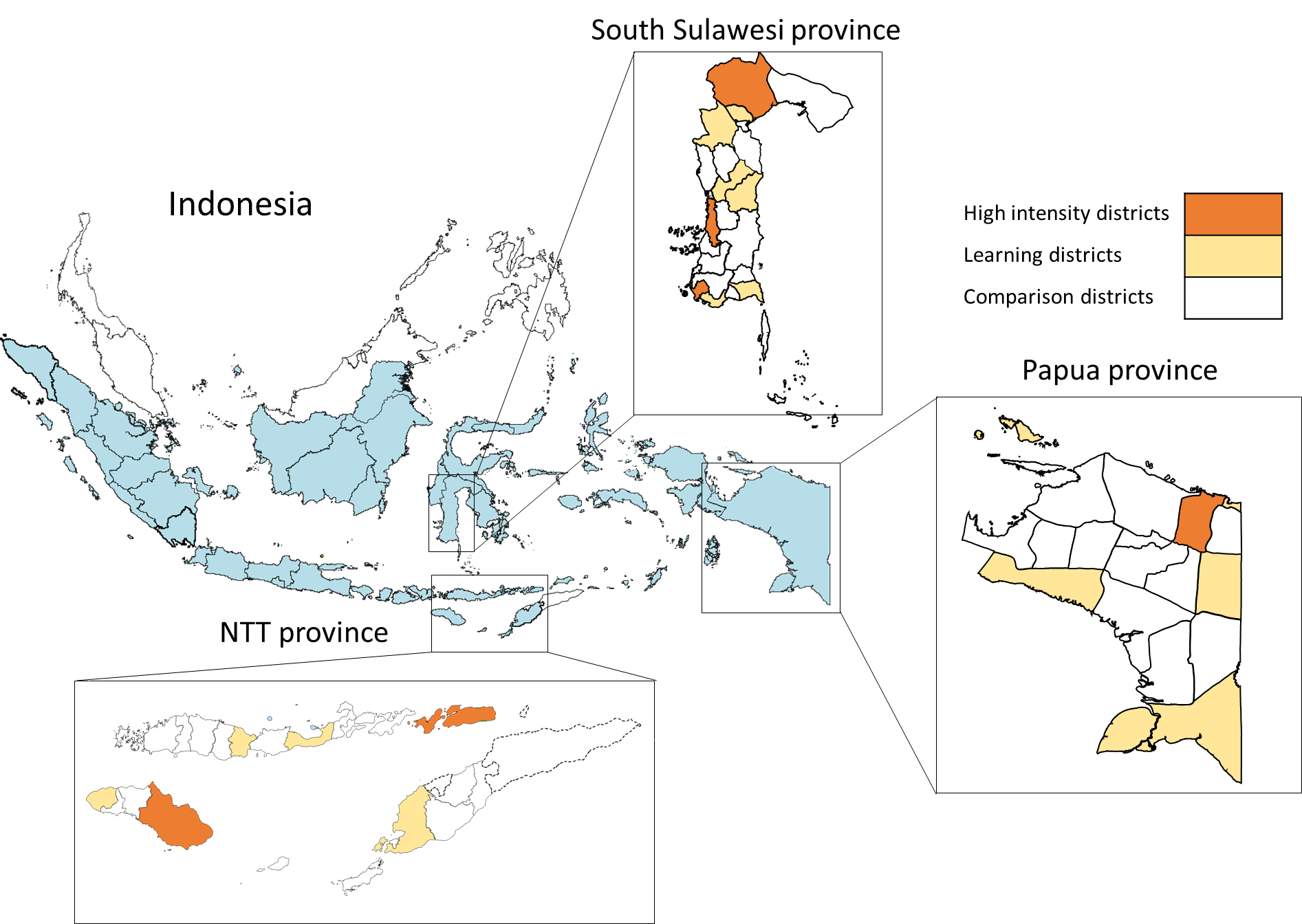


2. Approaches for primary outcome estimation and assumption

*Coverage of toilets built in “high-intensity” and “learning” districts:* STBM-SMS based data and SUSENAS are different data collection mechanisms, i.e. program reporting (STBM-SMS based data) vs. survey approaches (SUSENAS). The tracked indicators (i.e. definition of sanitation facilities) are also different. For STBM-SMS based data, there were limited number of villages with data already entered into the system at the beginning of the program, resulting in challenges to calculate the actual baseline number of toilets. Therefore, we selected villages recorded in the system (i.e. data was available) for July 2014 (South Sulawesi), October 2014 (NTT) and January 2015 (Papua) and used these as a baseline to estimate the increase in number of latrines from the baseline to June 2017 in these selected villages. These villages used to estimate a baseline in “high-intensity” and “learning” districts account for 30.0% and 30.7% of total villages, respectively. To estimate the total increase in all villages, the results were extrapolated to 100%, assuming the increase in these villages reflect similar changes across the remaining villages in the program areas. The SMS-based monitoring system collects indicators, including the number of households using permanent hygienic toilets (known as *Jamban Sehat Permanen* - JSP), and semi-permanent hygienic toilets (known as *Jamban Sehat Semi Permanen* - JSSP) defined by MoH. Total number of JSP + JJSP was reported as the number of toilets.

3. Approaches for key performance indicators (KPIs) estimation and assumptions

*Sustained usage: Slippage rate in ODF communities 1 - 3 years after verification:* Slippage rates were estimated for the endline survey in 6 high-intensity districts (24 ODF villages) in August 2017 (AAN & UNICEF, 2017). Results were analysed to estimate slippage rates in ODF villages verified as ODF for a minimum period of 1 to 3 years. The number of households sampled per village ranged from 58 to 140. Latrine usage was measured via a combination of direct observation (i.e. latrine use at household-level) for those who own a private latrine, and self-reporting by respondents for those who do not own a private toilet (i.e. self-reported open defecation practice most of times). For direct observation of household-level latrine use, six signs including (1) path to a latrine is walked on, (2) visibly used anal cleansing material observed, (3) if pour flush latrine, water is available, (4) detected faeces in pit using flashlight, (5) slab is wet, and (6) smell in toilet, were observed by enumerators. A latrine was considered being used by the household when at least one sign was observed at the time of survey.

*Reaching the poorest in high-intensity districts (among poorest 40% nationally):* This indicator in “high-intensity” districts was estimated from SUSENAS with the sample size designed to be representative at district level. The poorest 40% wealth quintiles nationally were calculated based on annual expenditure per capita. The proportion of the poorest 40% households owning a private latrine increased from 67.1% (2013) to 75.2% (2017), respectively. Given that SUSENAS has limitations around wealth quintile estimates (i.e. basis of annual expenditure per capita and fluctuations of each wealth quintile population estimate across different years), endline survey results were also analysed to examine this KPI. Private latrine ownership in the beginning of the program (August, 2013 as a baseline condition) was retrospectively estimated by asking respondents when they built a latrine, while latrine use is defined as households owning a private latrine or reporting to use a shared latrine. It was assumed that most households without a private latrine did not use a shared latrine before the program started. A principal component analysis (PCA) was performed for wealth score calculation, and households were classified into wealth quintiles in two ways; wealth quintiles in surveyed villages, and wealth quintiles adjusted for those falling into the poorest 40% of Indonesia as opposed to the poorest 40% of the local area. On this basis, 53% of households in high-intensity districts are in the poorest 40% of Indonesia, based on the national poverty thresholds calculated by SUSENAS.

*% of triggered communities (high-intensity districts) claimed or already verified ODF by end of the program:* STBM-SMS based data were used for KPI#5 estimation. STBM-SMS based data capture ODF villages (with the village, or desa in Indonesiam, as the unit of measurement) given that ODF verification is conducted at village-level.

*Leveraged resources: Number of project district plans with budgets for scaling up the program as a result of UNICEF advocacy:* Through Province Health Office (PHO) and District Health Office (DHO) administrative documents and UNICEF monitoring, it was confirmed that all high-intensity and learning districts in South Sulawesi and NTT, and three districts in Papua (Jayapura, Biak and Kota Jayapura) budgeted for STBM implementation and scaling up.

*Innovation and diffusion: Number of non-project districts with plans to implement STBM (CATS)* as a results of UNICEF advocacy: Available government data were collected for this indicator, and we report a total of 28 districts (South Sulawesi: Bantaeng, Enrekang, Gowa, Maros, Pangkep, Sinjai, Soppeng and Selayar, NTT: all districts, and Papua: Boven Digoel, Jayawijaya, Nabire and Keerom) having plans to implement or scale up STBM as a result of different levels of UNICEF advocacy. UNICEF support to these districts is summarized below.

Types of UNICEF support and contributions to non-UNICEF support districts vary between provinces. In South Sulawesi, it was confirmed that all 21 districts allocated budget for STBM implementation/scale-up in 2016 or 2017 in a provincial Pokja AMPL meeting. UNICEF provided technical support to all districts for strengthening an STBM-SMS based monitoring system through PHO. In a knowledge sharing event, with UNICEF support, two program districts (Luwu Utara and Wajo) shared key STBM achievements and learning with all districts in South Sulawesi. UNICEF also supported the organization of a Knowledge Management fair for enhancing cross-learning between districts. Seven non-UNICEF supported districts (Bantaeng, Enrekang, Gowa, Maros, Pangkep, Sinjai, and Soppeng) together with program high-intensity and learning districts presented their achievements (i.e. STBM implementation strategies, budget and regulations) in the event. UNICEF also provided technical advice around acceleration of STBM to Selayar District (based on their request), which UNICEF had previously supported between 2008 to 2013.

In NTT, with UNICEF support, PHO has developed a key strategy to accelerate STBM implementation in all districts, which is expected to influence district planning and budgeting for STBM implementation/acceleration beyond UNICEF supported areas. UNICEF also supported Districts via the Android M&E app being rolled out by the Province Pokja AMPL.

In Papua, Boven Digoel, Jayawijaya, Nabire and Keerom have started allocating budgets for STBM implementation after this program was initiated. PHO invited these districts for STBM workshops where UNICEF provided technical support. UNICEF also invited them for several STBM activities such as a cross-learning trip in which they participated using their district budget. Looking at a broader influence within Papua, UNICEF supported PHO to adopt two indicators, "N of ODF villages" and "N of villages where STBM is implemented", for tracking district performance. This will strengthen PHO oversight mechanisms and help PHO identify under-performing districts.

4. Financial analysis

A financial analysis was undertaken of the 4 main sources of funds used in the program, namely BMGF funds, additional funds from UNICEF, Govt funds (APBD (Anggaran Pendapatan dan Belanja Daerah / Regional Budget (at Province or District level)) & dana desa (village fund)) and finally investment from households in the high-intensity and learning districts themselves. Investment from households was estimated based on the number of toilets built and average unit cost for building a toilet within district. The total program value was over $12 million, of which 41% came from the donor (BMGF), 14% from UNICEF, 14% from Government (high-intensity districts) but the greatest investment (44%) came from the households. Figure S1 shows the yearly trend from 2013 (when no District or Province level data was available – either not allocated or figure not found) to 2017 (January to July) and shows a steady increase in Government fund expenditure each year.

5. Results of statistical analysis

To explore associations between levels of support received across the three district groupings (i.e. high-intensity, learning and comparison districts) and the primary outcomes of interests including (1) proportion of ODF villages, and (2) proportion of newly constructed household toilets per district over the programme period (2013-2017). Observed rate/proportion was modelled by using the negative binomial with the offset variable (i.e. the number of villages/households within each district) and robust standard errors, accounting for clustering effects at district level. Table S2 and S3 summarizes the results.

**Table S2.** Effect of levels of intervention intensity on the number of open defecation free villages verified during the intervention period (2014 - 2017), calculated as rate ratio from a negative-binomial regression model with log link and robust standard errors with the total number of non-ODF villages within each district used as the offset variable.

| Tested variables | N of districts | Rate ratio | 95% Confidence Interval | | *P*-value |
| --- | --- | --- | --- | --- | --- |
|  |  |  | Lower | Upper |  |
| Intensity level |  |  |  |  | < 0.001 |
| High (direct districts) | 6 | 4.65 | 2.12 | 10.20 | < 0.001 |
| Medium to low (indirect districts) | 16 | 1.75 | 0.94 | 3.27 | 0.08 |
| None (other districts) | 53 | Ref. | - | - | - |
|  |  |  |  |  |  |
| Province |  |  |  |  | < 0.001 |
| South Sulawesi | 24 | 18.68 | 7.97 | 43.79 | < 0.001 |
| NTT | 22 | 26.80 | 11.41 | 62.98 | < 0.001 |
| Papua | 29 | Ref. | - | - | - |
|  |  |  |  |  |  |
| Proportion of people living below poverty line in percentage | 75 | 0.94 | 0.90 | 0.97 | 0.001 |
|  |  |  |  |  |  |
| Proportion of ODF^a^ village at baseline in percentage | 75 | 1.02 | 1.00 | 1.04 | 0.02 |

^a^ ODF: Open defecation free

**Table S3.** Effect of levels of intervention intensity on the number of newly constructed toilets during the intervention period (2014 - 2017), calculated as rate ratio from a negative-binomial regression model with log link and robust standard errors with the total number of households within each district used as the offset variable.

| Tested variables | N of districts | Rate ratio | 95% Confidence Interval | | *P*-value |
| --- | --- | --- | --- | --- | --- |
|  |  |  | Lower | Upper |  |
| Intensity level |  |  |  |  | 0.059 |
| High (direct districts) | 6 | 11.15 | 1.04 | 119.82 | 0.047 |
| Medium to low (indirect districts) | 16 | 0.97 | 0.30 | 3.10 | 0.959 |
| None (other districts) | 53 | Ref. | - | - | - |
|  |  |  |  |  |  |
| Province |  |  |  |  | 0.009 |
| South Sulawesi | 24 | 14.52 | 1.99 | 105.93 | 0.008 |
| NTT | 22 | 9.39 | 2.08 | 42.43 | 0.004 |
| Papua | 29 | Ref. | - | - | - |
|  |  |  |  |  |  |
| Proportion of people living below poverty line in percentage | 75 | 0.98 | 0.91 | 1.06 | 0.648 |
|  |  |  |  |  |  |
| Proportion of OD^a^ households at baseline in percentage | 75 | 1.03 | 0.99 | 1.07 | 0.154 |

^a^ OD: Open Defecation
